# What impact do Global Health Initiatives have on human resources for antiretroviral treatment roll-out? A qualitative policy analysis of implementation processes in Zambia

**DOI:** 10.1186/1478-4491-7-8

**Published:** 2009-02-10

**Authors:** Johanna Hanefeld, Maurice Musheke

**Affiliations:** 1 Health Policy Unit, London School of Hygiene and Tropical Medicine, University of London, London, UK; 2 Zambia HIV related TB project (Zambart), University of Zambia, Lusaka, Zambia

## Abstract

**Background:**

Since the beginning of the 21^st ^century, development assistance for HIV/AIDS has increasingly been provided through Global Health Initiatives, specifically the United States Presidential Emergency Plan for AIDS Relief, the Global Fund to Fight HIV, TB and Malaria and the World Bank Multi-country AIDS Programme. Zambia, like many of the countries heavily affected by HIV/AIDS in southern Africa, also faces a shortage of human resources for health. The country receives significant amounts of funding from GHIs for the large-scale provision of antiretroviral treatment through the public and private sector. This paper examines the impact of GHIs on human resources for ART roll-out in Zambia, at national level, in one province and two districts.

**Methods:**

It is a qualitative policy analysis relying on in-depth interviews with more than 90 policy-makers and implementers at all levels.

**Results:**

Findings show that while GHIs do not provide significant funding for additional human resources, their interventions have significant impact on human resources for health at all levels. While GHIs successfully retrain a large number of health workers, evidence suggests that GHIs actively deplete the pool of skilled human resources for health by recruiting public sector staff to work for GHI-funded nongovernmental implementing agencies. The secondment of GHI staff into public sector facilities may help alleviate immediate staff shortages, but this practice risks undermining sustainability of programmes. GHI-supported programmes and initiatives add significantly to the workload of existing public sector staff at all levels, while incentives including salary top-ups and overtime payments mean that ART programmes are more popular among staff than services for non-focal diseases.

**Conclusion:**

Research findings suggest that GHIs need to actively mediate against the potentially negative consequences of their funding on human resources for health. Evidence presented highlights the need for new strategies that integrate retraining of existing staff with longer-term staff development to ensure staff retention. The study results show that GHIs must provide significant new and longer-term funding for additional human resources to avoid negative consequences on the overall provision of health care services and to ensure sustainability and quality of programmes they support.

## Background

There is a shortage of human resources for health (HRH) throughout sub-Saharan Africa [1]. Many countries in the region are also experiencing significant HIV epidemics, with an estimated 2.12 million persons needing antiretroviral medicines [2]. The lack of adequate human resources for health directly affects countries' ability to provide antiretroviral treatment to their population [3]. The disease burden of HIV and HIV-related mortality among health sector staff has further reduced human resources [4], at a time when the introduction of antiretroviral treatment in the public health system has substantively increased the workload of staff [5] and created an urgent need for additional human resources [6,7].

Strategies to address human resource deficits have centred around staff retention (through incentives such as allowances, salary top-ups, and better working conditions) and retraining, including shifting as many tasks as possible away from doctors, nurses and pharmacists to non-clinical staff, enabling clinical staff to concentrate on their specific areas of expertise [3,5,7]. In Malawi for example, where special attention has focused on addressing the shortage of human resources for health, all health sector workers have received a salary top-up to increase staff motivation, financed by funding provided to the Malawian Ministry of Health [8].

Many of the countries heavily affected by HIV and AIDS, which are facing a human resource crisis, are receiving large amounts of donor funding, including support for the large-scale provision of antiretroviral treatment through the public sector and private sector. Since the beginning of the 21^st ^century, development assistance for HIV and AIDS has increasingly been provided through partnerships and Global Health Initiatives (GHIs), specifically the United States Presidential Emergency Plan for AIDS Relief (PEPFAR), the Global Fund to Fight HIV, TB and Malaria and the World Bank Multi-country AIDS Programme [9].

Evidence of the impact of GHI programmes on human resources at country level, especially at subnational level, is limited. However, some studies have examined their impact in Ethiopia [10], and in Uganda, Mozambique and Zambia [11,12], and research findings are forthcoming from studies in Malawi and other countries[13].

This paper examines the impact of GHIs on human resources for ART roll-out in Zambia, at national, province and at micro level in two districts. The focus is on GHI's ability to contribute to retain and retrain staff, and also on unintended consequences of their programmes on human resources for health.

## Methods

The paper draws on more than 90 in-depth interviews with policy-makers and implementers at national and subnational level, engaged in processes governing the implementation of ART roll-out. Actors interviewed include national, provincial and district representatives from government institutions; the donor community; governmental and nongovernmental service providers; doctors and nurses; NGOs supporting the roll-out; programme managers; community workers; and networks of people living with HIV/AIDS.

Interviews were conducted in Zambia between August and December 2007, as part of wider, comparative research on policy processes relating to the implementation of ARV roll-out at national, provincial and district level. Interviews were conducted at national level, as well as at provincial level in one province, and district-level research was conducted in two districts within the focus province. Interviewees were selected based on a "snowballing" process originating from an in-country advisory panel, made up of academics, representatives of nongovernmental organizations, a Zambian clinician and a representative of a network of people living with HIV/AIDS.

Interviews were semistructured and used an interview guide that was tested and revised in consultation with the in-country advisory panel. Actors were interviewed about their perception of implementation processes relating to ART roll-out, as well as their role and personal history in relation to these processes. Where permission was granted, interviews were recorded and transcribed; otherwise extensive notes were taken.

A subset of 32 interviews was selected for this paper in which interview content focused on both GHIs and human resources. Interviews were analysed to identify five key themes identified: training, "top-ups", mentoring, coordination and recruitment of staff.

The research conducted is qualitative, so relies on, and is limited to, the perceptions of persons interviewed at national level, in one province and two districts, who are working in the ART roll-out and interacting with GHIs regularly in their work. To better understand the perceptions of actors at different levels, the results and discussion section highlight at which level – national, province or district – interviewees operate.

Where possible, the paper draws on available secondary research and data on human resources obtained by the authors during the research, allowing for validation of data collected. Given the recent, unfolding nature of the ART roll-out, and the limited secondary data available, this paper provides an empirical, contemporary spotlight on an underresearched and changing area. The research for this paper was conducted as part of a "twinning" project between a Zambian researcher and a UK researcher. Ethical clearance for the research was granted by the ethics committees of the University of Zambia and the London School of Hygiene and Tropical Medicine.

## Results and discussion

### Human resources for health in Zambia

Zambia faces a severe shortage in human resources, exacerbated by the country's HIV epidemic – an estimated 1.2 million (17%) Zambians are currently living with the virus – with less than a third of the recommended doctor-patient ratio [14] to treat the population. But the shortage of human resources for health is not limited to doctors, nor are they in the shortest supply. The greatest need is for laboratory technicians, followed by pharmacists, doctors, nurses and data monitors [interview, national level, November 2007].

Other problems have also been identified. For example, there is a rapid turnover of staff, high staff absenteeism [15] and an unequal distribution of staff between rural and urban areas [16,17]. Ministry of Health data revealed that in 2006, 368 staff members joined the public health sector, while 380 left the sector, highlighting a continued loss [15]. The main causes of attrition of health workers in 2004 were death and resignation of workers from the health service [16]. High vacancy rates of health posts throughout the public sector are well documented [14,15].

The human resource crisis is particularly urgent in relation to the ART roll-out, given the complexity of ART. Medicines need to be taken daily for the remainder of a person's life, and patients need to be initiated on the medication and reviewed on a regular basis by a doctor. Patients are also counselled by either a lay counsellor or a nurse on the importance of adherence to the treatment regime and a healthy lifestyle, while drugs need to be ordered and administered by a pharmacist. Despite the constraints, Zambia has had remarkable success in scaling up access to ART in the public sector. Between 2003 and the end of 2007, more than 130 000 persons were initiated on antiretrovirals out of 250 000 to 300 000 who are estimated to need such medication [interview, national level; October 2007].

To address the shortfalls in human resources, the Zambian government developed a specific human resources strategy in 2005, which has since received support from different donors. At the time this research was conducted, however, the only targeted human resource intervention receiving donor support, including through PEPFAR funding, was the rural retention scheme. This includes incentives to attract doctors into rural areas, including better housing, a car and a cash allowance [14].

### GHIs in Zambia

Zambia receives significant amounts of funding for its HIV programme from three Global Health Initiatives: the United States Presidential Emergency Plan For AIDS Relief (PEPFAR); the Global Fund to Fight AIDS, TB and Malaria; and the World Bank Multi-country AIDS Programme (MAP). In 2006 PEPFAR money alone made up 63% of all funding for HIV in Zambia [18]. This was in addition to resources for HIV from the World Bank MAP and the Global Fund.

However, mapping the flow of funding provided by individual GHIs in support of the public ART treatment programme is difficult[19]. This is in part because much of the funding supporting public sector programmes is channelled through NGOs or other private institutions and not directly to the government. For example, a recent study revealed that less than 5% of all PEPFAR funding for Zambia in 2005 was received by the government [19]. In some cases it is difficult to differentiate expenditure between intervention areas, such as treatment, prevention or care. Data on actual expenditure, i.e. funding disbursed to recipients at the country level, is also not easy to obtain, since PEPFAR and the World Bank MAP, for example, do not publicly share this information [18].

Despite the limitations in detailed information, broad information on funding was obtained. Interviews with key stakeholders confirmed that the preponderance of funding for treatment roll-out in the public sector is through GHIs, even if this is provided in the form of technical support and not direct funding to the government. Through consulting recent planning documents, a Ministry of Health official responsible for planning the ART roll-out for 2008–2009 expected "50% to 52% of funding from PEPFAR, 34% from the Global Fund and 10% to 15% or so from other sources" [interview, national level, November 2007].

PEPFAR funding is not allocated through the Ministry of Health but instead to US and national subrecipients, who then provide a range of support for prevention, care and treatment to facility, district and provincial level. PEPFAR subrecipients are mainly NGOs, (but also academic, private sector and government institutions) and, as they essentially implement the PEPFAR programme, they are also referred to as PEPFAR implementers. The impact and forms of this support concerning human resources, specifically support provided for treatment roll-out, are explored later.

The World Bank MAP grant, while in part envisaged to support the Ministry of Health's procurement of ART [20], in practice supported other elements of the programme, including laboratory supplies [interview, national level, November 2007] [18]. Global Fund resources are directly received by the Ministry of Health and at the time of conducting this research were paying for the actual ARV medication.

### The study focus province and district

The shortage of human resources for health was evident in the two study districts. At the time of conducting this research, six public sector clinics in one of the focus districts provided treatment to a population of 363 734 (GRZ 2000) with a staff of three doctors, one pharmacist and a changing number of technical (also called clinical) officers and nurses. In the second focus district, with a population of about 450 000, two doctors rotated between five clinics providing ART. Since 2004 more than 4000 people have started ART in each of the two districts, in clinics run by the district, with no additional staff provided by the Ministry of Health for these services.

In the study focus districts and province, public sector roll-out of ART was supported by one PEPFAR implementing agency, while additional PEPFAR support was provided for a private hospital in one of the districts. Funding to the Ministry of Health for actual medication and laboratory equipment aside, World Bank MAP and Global Fund support in the study districts and province focused on non-clinical interventions. In terms of supporting the clinical treatment roll-out at subnational level, PEPFAR implementers emerged as the most visible presence during the period of this research.

### GHI's addressing the human resources for health shortage

While GHIs do not provide direct financial support for additional human resources in the public sector, their programmes address the shortage in human resources through training for health care workers and volunteers in all aspects required to support the treatment programmes. They also provide allowances such as overtime payments, "top-ups", or payments of expenses, especially for volunteer counsellors or treatment support workers.

PEPFAR-funded programmes also provide ongoing mentoring or technical support in health facilities. This refers to clinical staff employed by a PEPFAR implementing organization who support health facilities, such as clinics or hospitals, on a regular basis (for example, through visits about once a week) to discuss issues relating to the treatment programme. They assist with questions relating to clinical management of patients. The exact models for technical support vary. Some PEPFAR organizations have staff based at provincial level, others send support teams from the capital on a regular basis.

In addition, PEPFAR implementers pay for, or second, data entry clerks in health facilities they support. These clerks record the number of persons who receive ART. Data are reported to both the Ministry of Health and PEPFAR. Similarly, clinical care specialists have been employed by a PEPFAR-funded organization and seconded to the provincial health directorates in each of Zambia's nine provinces.

While each of these interventions aims to alleviate the human resource shortage in relation to ART, examining their impact at district and provincial level in detail suggests possible negative, unintended consequences. The following discusses each of these interventions in turn, based on the evidence emerging from interviews with key stakeholders.

### "Top-ups": the impact of incentives for health workers in ART delivery

PEPFAR-implementing organizations provide "top-ups" to public health care workers and community volunteers working on the ART programmes they support. "Top-ups" are either overtime payments for shifts worked in the ART clinic or transport costs for meetings for those working on PEPFAR-funded health programmes. These incentives go a long way in motivating public health workers to work in the ART clinic. All nurses interviewed as part of this research confirmed that among their colleagues the ART clinic is the most popular [interviews, district level, October and November 2007], and their enthusiasm was echoed by the observations of policy-makers that ARV clinics or programmes are liked by staff.

While this suggests that "top ups" are successful in motivating staff to work on the ART programme, it raises concerns about possible unintended consequences. A recent study conducted among health care workers in three Zambian districts found that on average only 7% of health workers who had delivered non-HIV services had received incentives, underlining the clear financial benefits arising from involvement in ART delivery and causing imbalances between different parts of the service [11].

Some interviewees were concerned about the distorting effect of such payments, diverting attention and resources from non-focal diseases [interview, national level, October 2007]. Evidence collected was not clear on whether or not this is the case in the day-to-day delivery of services at health facility level.

However, policy-makers and planners interviewed at national level felt strongly that their work had focused largely on HIV and related diseases, to the neglect of other equally urgent health issues. This may possibly be a reflection of the time and attention devoted at that level to coordination of these activities. One senior Ministry of Health official observed, "HIV, TB and malaria have taken almost 90% of our time, not to mention that they have also taken most of our budgetary money to the extent that we have actually neglected what we call noncommunicable diseases" [interview, national level, October 2007].

The provision of short-term incentives such as top-ups may also have implications for sustainability, including quality of care. Speaking about the effect on the quality of care in the longer term, a senior Ministry of Health official explained: "They [donors] support short-term incentives ...but those are highly unsustainable because they are applied for a year. You put so many people on treatment because you are providing services to the health worker, then the following year there is nothing ..." [interview, national level, November 2007].

What this official points to is the effect of the one-year funding cycle of PEPFAR, which means that incentives cannot be guaranteed beyond that time frame, which may create resentment among existing staff members, who narrowly miss out on receiving top-ups or change their performance from year to year. There may also be a negative impact on long-term quality of care if top-ups are withdrawn after a year, and this underlines concerns about sustainability of the programmes. An advisor to the Ministry of Health said: "They [GHIs] are going to leave everything flat when they leave" [interview, national level, November 2007].

This suggests that while top-ups or incentives are successful in motivating existing health care workers to work in the ARV clinics, they may have negative immediate consequences on attention paid to the quality of care provided to non-focal diseases. This echoes findings on the impact of top-ups by Ooman et al. [12]. As top-ups are not sustainable beyond the period funded by a GHI, it also raises concerns about the ability to sustain quality of care for patients in the longer term.

### Training and mentoring of health care workers for ART provision in Zambia

One of the key GHI elements of support is training for health care workers. As a clinician from a PEPFAR implementing organization described their strategy: "We put money into doing additional training for clinical officers, medical officers...and if their sites are growing rapidly and they need additional training, the team goes and assesses the needed training" [interview, national level, October 2007].

The training helps build capacity of health care workers involved at different levels in the provision of ART. However, health care workers often leave the public sector or their position once they are trained. All PEPFAR implementing agencies supporting the ART roll-out in Zambia described this as a common experience and a key challenge. A senior district health official replied, when asked about the greatest challenge faced in implementing the ART roll-out: "human resources ...you train people to provide this and within a short time they have left. So you need to find people to continue providing the service. That has been a major challenge in terms of implementing..." [interview, district level, September 2007]. The very fast turnover of staff once trained suggests that external training, in isolation from increased resources to enable career progression and longer-term incentives in the public sector, has little effect in alleviating the shortages of skilled health care workers to support the provision of ART.

In addition, training, especially the per diems provided during such training, are part of the reasons that attract health workers to work on the ART programme, adding to the potentially distorting effects of top-ups. A further consequence of training, when externally conducted, means that these are short-term, intensive courses that take clinicians out of their clinic, imposing a further strain on the day-to-day running of the ART programme.

### Mentoring and the secondment of GHI staff to the public sector

In addition to training, the PEPFAR implementing organizations in Zambia supporting ART roll-out provide ongoing technical support through mentoring of health care workers. This involves visiting ART sites and attending to patients together with health care staff, to monitor the quality of services and assist with difficult clinical cases. Some organizations have teams of specialists, ranging from clinical care to pharmacy, nursing and logistical support, who visit the clinics and hospitals supported by their organization on a monthly or weekly basis.

In some cases, technical support is provided by staff recruited and employed by a PEPFAR implementing agency and seconded to the public health sector, where they work alongside their public sector colleagues. An example of this practice is the PEPFAR-funded Health Services and Systems Programme (HSSP), aimed at providing technical support to the Ministry of Health. It focuses on aspects relating to the health systems and human resources.

As part of its support, HSSP recruited and seconded clinical care specialists to each of Zambia's nine provincial health directorates, to provide technical support to the districts and hospitals in the delivery of ART services [Abt Associates, 2007]. These clinical care specialists work in the provincial health directorate alongside a clinical care specialist employed by the Ministry of Health, but receive a higher salary. While part of the provincial health team, they also have access to a small operational budget for training and ongoing support [interview, national level, October 2007]. All nine clinical care specialists employed by HSSP are physicians, whereas the government's counterparts are nurses or technical or clinical officers (holders of a three-year, diploma-certified degree that in Zambia allows clinical practice).

While these clinical care specialists are in addition to the provincial team and undoubtedly contribute through their skills and commitment, given the salary level and remit, these posts are not sustainable beyond HSSP funding. In addition, their relative seniority compared to the government's clinical care specialists raises questions about working (and status) relationships that may affect, both positively and negatively, the implementation of services. Some national actors reported that the clinical care specialists had led to an increase in capacity, while implementers at provincial and district level reported that their engagement may have led to demotivation of government staff. In addition, interviews suggested that nurses and technical officers at district level referred to the MoH clinical care specialist, whereas doctors worked with the HSSP-employed clinical care specialist.

### Increasing workload through coordination

Despite efforts at national level to coordinate activities between the different implementing partners and the Zambian government through a range of bodies, including technical committees that determine a geographical and skills-based division of labour, policy-makers interviewed mentioned that coordination with individual organizations remained problematic. A senior official at the Ministry of Health said: "In Lusaka alone there are close to 236 partners working on HIV...to track what they are doing is a challenge" [interview, national level, November 2007]. When describing the coordination, another Ministry of Health official said: "...it is overwhelming, there is a lot that needs to be done and sometimes I feel as if am doing injustice to some of the activities" [interview, national level, November 2007].

There is clear evidence, from the data collected and other research focusing on human resources and ART, that the workload for staff has increased since the introduction of ART [4]. In one study district, the same number of doctors and nurses as in 2004 (before district provision of ART) were providing treatment and care to more than 4000 patients on ART by the end of 2007 [interview, district level, October 2007]. As previously highlighted, the district staffing levels at the time of conducting this research were two and three doctors in clinical care, respectively, in the focus districts, and approximately 10 members of staff at the provincial health administration. Health care workers interviewed said that their workload had not only increased due to a greater number of patients, but also due to coordination of activities funded or implemented by GHIs.

At province and district level, the coordination with PEPFAR implementers posed an additional workload for health sector staff, due to funding requirements. Districts supported by PEPFAR in their roll-out were required to provide monthly reports to the provincial office of the PEPFAR implementer. These were in addition to quarterly reports that form part of the Ministry of Health processes and the MoH's twice-annual performance reviews.

To streamline the process and avoid confusion, each district in the province had appointed a focal person to interact with PEPFAR implementers [interview, district level, October and November 2007]. Focal persons were drawn from among doctors, nurses and clinical officers working at the district level.

PEPFAR implementers held quarterly meetings with supported districts to review activities. In addition, PEPFAR implementers supported the district teams to have further regular meetings, to either coordinate with other stakeholders, such as NGOs, or to discuss issues of clinical management.

While PEPFAR implementers provided resources for these meetings, their organization and arrangements are the responsibility of the district focal person, in addition to his or her clinical workload. The rationale for making this a district responsibility was to ensure that the district managed the programmes in an integrated way. However, there were opportunity costs to district staff – such as time. Meetings tended to last a whole working day. As one district staff pointed out: "Our work has increased, like when it comes to meetings, I have to write the memos, to contact people...we have about three meetings in a month clinical meeting, quarterly review meeting and the quarterly referral meeting – which usually take the whole day..." [interview, district level, November 2007].

Many new initiatives instigated and supported by GHIs, including through financial resources for training and materials, must be implemented at existing staff level. An example of this is the introduction of ART site accreditation, introduced with the technical assistance of a local PEPFAR implementer who helped develop a standard set of indicators against which to assess sites' readiness to be accredited to have minimum requirements in place, for the provision of ART. The Zambian Medical Council has been designated to oversee the process, receiving a minimal budget for overseeing and facilitating the accreditation process, by use of existing funds for monitoring and evaluation [interview, national level, November 2008].

At provincial level, the accreditation of sites, which involves a site visit and assessment, is conducted through teams that draw on existing provincial health administration staff (10 persons) and medical practitioners from the provincial hospitals. By mid 2007, more than 30 sites were already providing ART in the focus province, and as accreditation of sites was introduced several years after the start of the public sector ART programme, these sites needed to be assessed. This was in addition to any new sites for ART roll-out [interview, district level, October 2007].

Accreditation requires a site visit and assessment. Given staffing levels, with no additional human resources available for the accreditation processes these were understandably delayed [interview, national level, November 2008]. While site accreditation is undoubtedly an important element of quality assurance, the way in which this was introduced, and its implementation envisaged, shows the limitations of such initiatives in the absence of additional funding for human resources.

This suggests that support by GHIs, particularly PEPFAR implementers, is provided in the form of training and financial support for materials and meetings, for many new initiatives that may improve the ART programme and ensure greater quality of care and treatment. Despite the clear benefits of the intended outcomes, the lack of funding for additional human resources within the health sector adds significantly to the workload of already stretched human resources for health, risking further burnout and ultimately contributing to making programme efforts less sustainable.

### GHI recruiting

A further impact of GHIs on human resources for health is the actual recruitment of health workers from within the public sector, by the various implementing agencies of GHIs, especially those funded through PEPFAR. This is particularly apparent in the support provided for a clinical intervention, such as the provision of ART roll-out, where assistance, including training and mentoring, requires clinicians familiar with the Zambian health system.

Of 15 health workers (including doctors, nurses and pharmacists) currently working for GHIs or their implementers who were interviewed for this study, nine had recently been recruited from the public sector. One senior Ministry of Health official described how PEPFAR agencies recruit government employees once they have gained experience, and then describe the government as lacking capacity: "It [PEPFAR] is strategically weakening government efforts....What is happening is that we are training people ...next you will hear that he has been taken ...next you will hear that government, you have no capacity" [interview, national level, December 2007].

Of the health workers involved in the two public sector sites that started ART in Zambia in 2002 (University Teaching Hospital, Lusaka, and Ndola Central Hospital), including the doctors leading these programmes, the majority have now left the public sector to work for GHI-funded organizations that support the roll-out of ART [interviews, national level; September-November 2007]. It appears that GHIs, by recruiting local health care workers to provide the technical support for ART, are drawing precisely from, and depleting the pool of, the most-qualified health workers in Zambia. These findings corroborate the practices observed in a three-country study by Ooman et al. [12].

## Conclusion

Global Health Initiatives have vastly expanded access to life-saving treatment for thousands of people in Zambia, yet they are not effectively addressing the human resources for health shortages in their programmes supporting ART roll-out. Given the overall amount of their resources aimed at supporting ART programmes, comparatively little is being done to address the health worker shortage. While some of their interventions, such as top-ups for staff working on ART, secondment and training appear to alleviate staff shortages in the short term, and succeed in giving many health sector staff the opportunity to improve their knowledge and skills on HIV/AIDS through short term training, workshops and on-the job training, they appear less successful at staff retention. This echoes similar findings from three further districts in Zambia recently published [11].

GHIs' programmes have increased the workload of already-stretched managers and health care providers. As the majority of GHIs, particularly PEPFAR, support treatment through individual organizations, such as NGOs, there is a significant added workload for public sector health staff who have to coordinate these support activities. This appears to be the case at all levels from national to district level, adding to potential problems of staff burnout. The recruitment by GHIs of public sector health workers to work for GHI-funded nongovernment implementers that support public sector roll-out further reduces the human resources for health in the public sector in Zambia. It also raises concerns about the ethical dimension of this assistance, where instead of providing much-needed resources to the government to increase human resources for health, development agencies use aid money to hire public sector workers to provide external assistance to the ART programme.

When recommending and supporting new policies, such as site accreditation for ART, GHIs should conduct a human resource impact assessment and address the human resource needs created by such interventions through additional funding that will allow the government to recruit the staff required to implement them through the public sector.

By not providing resources for the MoH to employ further human resources, but seconding them, as in the case of additional provincial clinical care specialists, additional capacity remains external and limited to the period of GHI funding available. The more Zambia's treatment programme relies on mentoring and seconded staff, the less sustainable it becomes in the long term, creating greater dependence on GHIs to continuously fill these gaps in capacity. Different approaches, such as the model followed in Malawi, should be explored to avoid creating further dependence [8].

Similarly, as training is provided through external partners and not integrated into a longer-term strategy for developing the human resources and allowing individuals to progress professionally within the health system, there are limited incentives for health professionals to remain within the public health care system.

The impact of top-ups increases staff motivation and interest in the ART programmes, but may have a distorting effect on health services overall. There is concern that as staff move vertically towards the ART programme, quality of services for non-focal diseases may suffer. In addition, the short-term nature of funding cycles may mean a drop in quality of care for patients on ART, once these payments are discontinued. More research is needed to assess the impact of top-ups for disease-specific programmes on the overall provision of health care services.

These interventions, aimed at addressing the shortages in human resources for health, including top-ups, mentoring, secondment of staff and training, all appear "surgical" in that they are not genuinely interwoven into the Zambian health system at all levels. They could be removed or abandoned, leaving a nearly hollowed-out treatment programme behind.

The evidence discussed in this paper – from interviews with Zambian health workers at all levels from the national Ministry of Health to districts and clinics – suggests that GHIs need to rethink the impact of their overall programmes, policies and conduct in relation to human resources for health. They need to address the long-term effect on quality of care and health systems of interventions targeted at alleviating staff shortages to avoid creating an ever-growing dependency of the Zambian treatment programme on external actors.

## Competing interests

The authors declare that they have no competing interests.

## Authors' contributions

JH conceived the study and its design. MM and JH conducted interviews jointly, and worked together on transcribing and analysing data collected. They jointly developed an outline for the paper and wrote the initial draft, which they revised following comments from reviewers. All authors have read and approved the final manuscript.

## Authors' information

Johanna Hanefeld is a PhD candidate at the London School of Hygiene and Tropical Medicine, researching policy implementation processes relating to ART roll-out in Zambia and South Africa. Maurice Musheke is a social scientist based at Zambart. This article is the result of a "twinning" between the two researchers.
